# Cost-effectiveness of a male catch-up human papillomavirus vaccination program in the Netherlands

**DOI:** 10.1016/j.pmedr.2022.101872

**Published:** 2022-06-27

**Authors:** Joost J.M. Simons, Tjalke A. Westra, Maarten J. Postma

**Affiliations:** a Market Access Department, GSK, Van Ash van Wijckstraat 55H, 3811 Amersfoort, the Netherlands; b Department of Health Sciences, University of Groningen, University Medical Center, Hanzeplein 1, 9713 GZ Groningen, the Netherlands; c Unit of Pharmacotherapy, -Epidemiology & -Economics, University of Groningen, Groningen Research Institute of Pharmacy, A Deusinglaan 1, 9713 AV Groningen, the Netherlands; d Department of Economics, Econometrics & Finance, University of Groningen, Faculty of Economics & Business, P.O. Box 800, 9700 AV Groningen, the Netherlands

**Keywords:** Cost-effectiveness, Modeling, HPV, Vaccination, Catch-up, Male, ICER, incremental cost-effectiveness ratio, HPV, human papillomavirus, WTP, willingness to pay

## Abstract

In the Netherlands, the Health Council has advised that the human papillomavirus (HPV) vaccination should be offered to both boys and girls. Additionally, boys and men up to the age of 26 years should be included in a catch-up program. In this study, we examine the cost-effectiveness of this HPV catch-up program.

We used a static Markov model to estimate the amount of cancers prevented and the incremental cost-effectiveness ratio (ICER) for different scenarios.

Vaccinating men from 12 until the age of 26 years would result in an average of 48 cancer cases prevented in every cohort (an estimated total of 720 cases), with an average ICER of €32,256.

We found that the catch-up vaccination program results in a relevant number prevented cases against an acceptable cost-effectiveness ratio. Policymakers should take these findings into account when evaluating a gender-neutral HPV vaccination program in the Netherlands.

## 1 Introduction

Human papillomavirus (HPV) vaccination is available for Dutch girls since 2009 in the Netherlands. In addition to vaccinating 12-year-old girls, all girls aged 13–16 years were invited to receive the HPV vaccine in a catch-up program (Gefenaite et al., 2011).

**Fig. 1:**
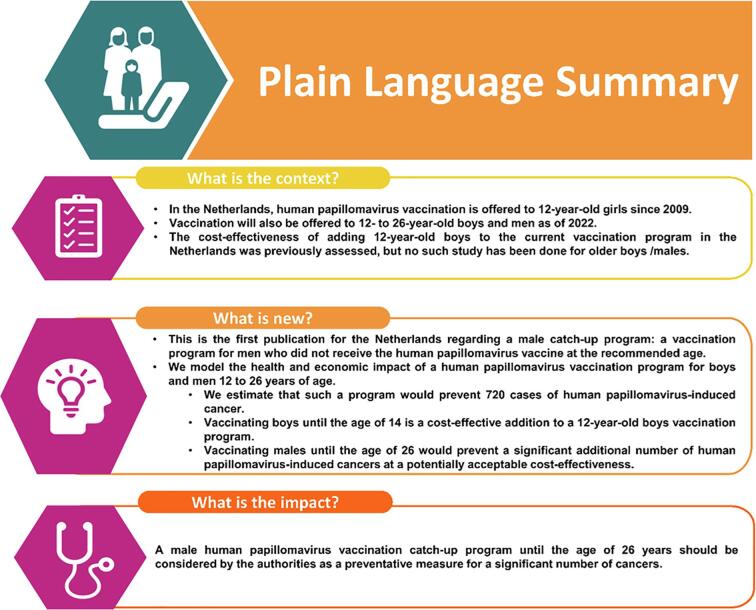

Since the introduction of the program in 2009, vaccination coverage in girls has been suboptimal reaching less than half the girls that are invited. Thus, the government decided to further expand the HPV vaccination program to boys, to further reduce the burden of HPV-related disease in the Netherlands (Fig 1).

In 2022, boys are planned to receive HPV-vaccination in the Netherlands (HPV-vaccinatie, 2021). The HPV vaccines have shown to be effective in the male population by providing (i) direct protection of boys for HPV-induced cancer at a later age, including penis, anus and oropharyngeal cancers, and (ii) indirect protection for girls due to reduced transmission (Harder et al., 2018, Elbasha et al., 2007).

There is still debate around whether men until the age of 26 should have the opportunity to receive the HPV-vaccine.

Previously, we showed that the vaccination of 12-year-old boys can be considered cost-effective (Simons et al., 2020). Here, we aim to assess the impact of a catch-up HPV-vaccination program for boys and men until the age of 26 years in the Netherlands from a healthcare-payer’s perspective.

## 2 Material and methods

### 2.1 Model characteristics

The developed model reflects a lifetime multi-stage static Markov approach with time cycles of one year, comparing HPV vaccination of one single cohort of 100,000 boys or men with the current situation of the female-only strategy. In this model, one cohort is vaccinated and followed until the age of 95. A full overview of the model, all of its assumptions and input parameters, have been previously described, and has been added in the Supplementary Materials (Simons et al., 2020).

The age of vaccination was varied between 12 and 26 years old based on the recent Dutch Health council advice (Health Council of the Netherlands, 2019). We assessed the number of cancers prevented and the incremental cost-effectiveness ratio (ICER’s) for all ages.

Additionally, multiple scenarios combining different age groups were assessed. These analyses estimate to what degree a catch-up program could be considered as (cost-)effective in preventing HPV-related cancers, and which age groups should be included.

We performed a multi-cohort analysis to assess the total cost of implementing a catch-up program.

We assessed three different scenarios. In the first scenario, boys aged 12–14 years are vaccinated. In the second scenario, boys aged 12–16 years are vaccinated, the same age range previously established in the girl’s vaccination scheme. Finally, we assessed the scenario in which boys aged 12–26 years are vaccinated. In all scenarios, boys aged 12–14 years receive two doses, and those aged 15 and older receive three doses. We assumed a vaccine coverage of 30% with a vaccine price of €50 per dose, which is 50% lower than the current listed price (Qendri et al., 2019), since vaccination will be offered within a tender based scenario. Vaccine efficacy data was based on clinical study data of the bivalent HPV vaccine from the PApilloma TRIal against Cancer In young Adults (PATRICIA) (Lehtinen et al., 2012, Paavonen et al., 2009). For the catch-up vaccination analyses, no herd immunity to the female population was assumed.

Finally, sensitivity analyses were performed to assess the impact of key parameters on the outcome of the model. Three deterministic sensitivity analyses (DSA) were performed at different ages (12, 18 and 26) to assess the impact of different variables, for example: female vaccination coverage, infection rate, vaccine cost and vaccine efficacy. An overview of the values used for the deterministic sensitivity analysis is shown in the Supplementary Materials. Additionally, three probabilistic sensitivity analyses (PSA) analyses were performed at the same vaccination ages (12, 18, 26), to assess the impact of varying multiple key parameters at the same time on the outcome of the model. For the PSA 1,000 simulations were used.

## 3 Results

*In the base case, vaccinating 12-year-old boys prevented 56 cases of HPV-induced cancer, with a corresponding ICER of €17,907. When increasing the vaccination age, the number of prevented cancers declined, and conversely, the ICER increased. At age 26 years, the number of HPV-induced cancers that were prevented over the remaining lifetime decreased of approximately 40%, to 32 cases, with a corresponding ICER of €53,173.*

Table 1 shows the number of HPV-cancers prevented and the corresponding ICER of all vaccination age scenarios ranging from 12 to 26 years old in the base case analysis.

**Table 1:** Impact of multi-cohort vaccination in the Netherlands.

Vaccination age (years)	Cancer cases prevented per vaccinated cohort	QALY gained per vaccinated cohort*	Cost difference vaccination vs no vaccination	ICER per vaccinated cohort
12	56	205	€3,672,920	€17,907
13	55	200	€3,675,342	€18,342
14	55	200	€3,675,346	€18,343
15	55	200	€5,589,748	€27,925
16	55	199	€5,559,116	€28,027
17	53	192	€5,594,044	€29,169
18	52	189	€5,595,361	€29,575
19	51	184	€5,597,818	€30,361
20	49	176	€5,601,928	€31,771
21	47	166	€5,607,374	€33,850
22	44	155	€5,612,999	€36,296
23	42	143	€5,618,746	€39,182
24	39	132	€5,624,836	€42,774
25	36	119	€5,631,014	€47,147
26	32	106	€5,637,896	€53,173

ICER: incremental cost-effectiveness ratio; QALY: quality-adjusted life year *rounded, ICER is based on non-rounded QALY.

### 3.1 Multi-cohort analyses

In the first scenario, the model predicted that vaccinating boys aged 12 to 14 years would prevent 166 cancer cases. On average, this resulted in 55 cases per vaccinated cohort and an ICER of €18,197.

In the second scenario, vaccinating boys aged 12 to 16 years prevented 275 cancer cases with an average ICER of €22,109.

In the final scenario, boys and men until the age of 26 years were vaccinated in the model. This resulted in a total of 720 cases of HPV-induced cancer prevented and an average ICER of €32,256. An overview of all scenarios is shown in Table 2.

**Table 2:** Multi-cohort analysis.

Vaccination age (years)	HPV vaccine doses needed	HPV-related cancer cases prevented - Total	HPV-related cancer cases prevented – Average per cohort	QALY gained – Total *	QALY gained– Average per cohort*	Average cost difference vaccination versus no vaccination	ICER vaccination strategy vs no male vaccination
12	60,000	56	56	205	205	€3,672,920	€17,907
12–14	180,000	166	55	606	202	€3,674,536	€18,197
12–16	360,000	275	55	1005	201	€4,434,494	€22,109
12–26	1,260,000	720	48	2568	172	€5,219,633	€32,256

ICER: incremental cost-effectiveness ratio, HPV: human papillomavirus, QALY: quality-adjusted life year. The ICER is based on the average ICER of age cohorts and thus based on non-rounded QALY estimates. *Rounded.

### 3.2 Sensitivity analyses

#### 3.2.1 Deterministic sensitivity analysis

In the DSA, the ICER was found to be most sensitive to the degree of herd immunity from the female population in all three age groups, see Supplementary Materials: Figure S1). This is followed by the infection uncertainty and the cost per dose of the vaccine.

#### 3.2.2 Probabilistic sensitivity analysis

The scatterplot with the simulations from the PSA is shown in Supplementary Materials: Figure S2 of the three different age groups.

## 4 Discussion

These results show that a catch-up program for boys and men until the age of 26 years can be a nearly cost-effective addition to the boys *plus* girls’ vaccination program. The most favorable ICER, €18,197, is achieved by vaccinating all boys until, and including, the age of 14 years. Implementing an HPV vaccination program for all boys 12 to 16 years of age (the same age cohort used in the previous vaccination program for girls) would result in an ICER of €22,109, which is also considered to be a nearly cost-effective strategy, based on a willingness to pay (WTP) threshold of €20,000. Including HPV vaccination until the age of 26 years, would result in an ICER of €32,256. However, the projected ICER only included HPV-related cancer prevention in males. Including female cancer prevention due to herd immunity would improve the ICER significantly, as was seen in previous published studies (Marra et al., 2009), making vaccination until the age of 26 years potentially cost-effective. Adding boys/men to the vaccination program increases the vaccination program effectiveness as it extends benefits to non-vaccinated females. This is especially true in the Netherlands where the female vaccination coverage has been suboptimal since the start of the vaccination program in 2009. Using a WTP threshold of €20,000 when preventing a serious disease, such as HPV-induced cancer, makes the cost-effectiveness debatable however, using a WTP threshold of €50,000 or €80,000, used for severe diseases, would result in all scenarios being cost effective. Finally, the analyses only assess the impact of the vaccination on HPV types 16/18. Taking other HPV types into consideration would also result in a more favorable ICER due to cross protection.

As expected, the later the vaccination is given, the fewer number of cancers are potentially prevented. However, a significant number of HPV-induced cancers can be prevented in the older age cohorts, thus suggesting the need to broadly implement HPV-vaccination.

This is the first study in which the cost-effectiveness of a male catch-up vaccination program in the Netherlands has been assessed.

One of the strengths of this study is the fact that a straightforward Markov model was used and therefore the results have relatively low uncertainty. A limitation of the model is that it was not set up in a dynamic way. As the vaccine coverage among girls fluctuates and the vaccination coverage among males are still highly uncertain, a dynamic model would introduce a high level of uncertainty. For that reason, a static model might provide more relevant outcomes as this type of model does not consider indirect vaccine effects. To prevent overestimation of the effect of male HPV-vaccination, a correction factor has been applied to the total amount of HPV-infections in males, to reflect the reduced transmission of HPV in the population thanks to female vaccination. Adding the effects of herd immunity from male vaccination to the female population, would likely improve the current ICER’s considerably. The sensitivity analyses show results that are comparable to our previously published analyses. The results are as expected. In the DSA, no notable differences were observed comparing the different age groups. In the PSA, the incremental QALYs were comparable between the 12 and 18 years old vaccinated group, the biggest difference here was the incremental costs due to an additional vaccine dose needed in this age group. The 26 years-old group showed lower QALY gains, explainable by the fewer amount of cancers being prevented in this group due to higher age.

Finally, the goal of these additional calculations was to assess the impact of adding HPV vaccination of males on top of the currently running female-only program. Adding female vaccination would overshadow the vaccination benefits in males and potentially overestimate the power of vaccination in males.

## 5 Conclusions

In conclusion, a catch-up vaccination program for males until the age of 26 is considered to be nearly cost-effective and should prevent a relevant number of HPV-induced cancers. Therefore, following the Dutch Health council recommendations, a catch-up vaccination program should be considered for implementation in the Netherlands (Health Council of the Netherlands, 2019). Supplementary Figure S3 presents a summary of the context, outcomes, and impact of this study for healthcare providers.

## CRediT authorship contribution statement

J.J.M. Simons: Conception, design of the study, model construction, data collection, data interpretation, conduct of the study, critically reviewing or revising the manuscript for important intellectual content, manuscript writing.TA. Westra: Conception, design of the study, data collection, data interpretation, conduct of the study, critically reviewing or revising the manuscript for important intellectual content. M.J. Postma: Conception, design of the study, data collection, data interpretation, critically reviewing or revising the manuscript for important intellectual content.

## Declaration of Competing Interest

The authors declare the following financial interests/personal relationships which may be considered as potential competing interests:

J.J.M. Simons and T.A. Westra are employed by the GSK group of companies. T.A. Westra holds shares in the GSK group of companies. M.J. Postma declares outside of the submitted work grants and personal fees from MSD, the GSK group of companies, Pfizer, Boehringer Ingelheim, BioMerieux, Novavax, BMS, Astra Zeneca, Sanofi, Novartis, Ingress Health, Pharmerit and IQVIA. M.J. Postma declares outside of the submitted work to hold shares from Health-Ecore (20%) and from PAG Ltd (100%). M.J. Postma declares outside of the submitted work financial support from Asc Academics as an adviser for this company. J.J.M. Simons, T.A. Westra and M.J. Postma declare no other financial and non-financial relationships and activities.
