# Supplementary material for: Cost-effectiveness of a male catch-up human papillomavirus vaccination program in the Netherlands
Source: Prev Med Rep. 2022 Jun 27;28:101872. doi: 10.1016/j.pmedr.2022.101872 (PMC9251567; doi:10.1016/j.pmedr.2022.101872)
Supplement: Supplementary data 1 [file mmc1.docx]

Supplementary Material

Cost-effectiveness of a male catch-up human papillomavirus vaccination program in the Netherlands.

Joost J.M. Simons,

Tjalke A. Westra, Maarten J. Postma

Preventive Medicine Reports

## **Supplementary Table S1 Model utilities**

| **Utilities** |  |
| --- | --- |
| Optimal | Age dependent 0.96 - 0.81 [1] |
| Anal cancer | 0.57 [2] |
| Anal cancer survivor | 0.87 [3] |
| Penile cancer | 0.79 [2] |
| Penile cancer survivor | 0.85 [4] |
| Oropharyngeal cancer | 0.58 [2] |
| Oropharyngeal cancer survivor | 0.75 [3] |
| Female scenario |  |
| QALY loss / Cervical cancer case | 3.95 [5] |
| QALY loss / Vaginal cancer case | 4.59 [5] |
| QALY loss / Vulvar cancer case | 3.28 [5] |
| QALY loss / Anal cancer case | 3.05 [5] |
| QALY loss / Oropharyngeal cancer case | 5.16 [5] |

*QALY, quality-adjusted life-year*

## **Supplementary Table S2 Model costs**

| **Costs** |  |  |
| --- | --- | --- |
| Vaccination costs per dose | Costs per dose of AS04-HPV-16/18 vaccine, only the vaccine cost itself. | € 50 [6-8] |
| Administration costs per dose | Productivity cost for vaccinating and giving informed consent. Added for every dose of AS04-HPV-16/18 vaccine. | € 13.81 [9] |
| Total cost for vaccinated boy | The total cost for the government to vaccinate one boy with AS04-HPV-16/18 vaccine, assumed in this model. Based on a two-dose vaccination scheme with tender-based pricing. | € 127.62 [10] |
| Average costs anal cancer | Average total cost of one case of anal cancer, considering different grading | € 5,460.25 [10] |
| Average costs anal cancer death | Total costs per anal cancer-related death are estimated as the average costs in the last year of life at the mean age at death. These costs were based on health insurance, home care, nursing homes and mortality data. | € 22,051.96 [10] |
| Average costs penile cancer | Average total cost of one case of penile cancer, considering different grading | € 4,368.20 [10] |
| Average costs penile cancer death | Total costs per penile cancer-related death are estimated as the average costs in the last year of life at the mean age at death. These costs were based on health insurance, home care, nursing homes and mortality data. | € 22,051.96 [10] |
| Average costs oropharyngeal cancer | Average total cost of one case of oropharyngeal cancer, considering different grading | € 6,552.30 [10] |
| Average costs oropharyngeal cancer death | Total costs per oropharyngeal cancer-related death are estimated as the average costs in the last year of life at the mean age at death. These costs were based on health insurance, home care, nursing homes and mortality data. | € 22,051.96 [10] |

*HPV, human papillomavirus*

## **Supplementary Table S3 Model assumptions**

|  | **Assumption** | **Source** |
| --- | --- | --- |
| 1. | No waning of vaccine-induced immunity | [11, 12] |
| 2. | Vaccine coverage comparable to 45.5% of the female coverage, set at 30% for boys. | [13] |
| 3. | 100% vaccine efficacy against HPV-16/18. No more infections with HPV-16 or HPV-18 are possible after vaccination. | [14] |
| 4. | 82.4% vaccine efficacy against infection with all non-HPV-16/18 types. Calculated based on the 100% efficacy against HPV-16/18 and the overall effectiveness of 93.2% against CIN3+. | [14] |
| 5. | 22% herd immunity in the male population due to the female-only vaccination program (+/- 45% coverage in female). This is varied in scenario analysis to assess the impact of increasing / decreasing female vaccination coverage. | [15] |
| 6. | At the time of vaccination (12 years old), no boys are infected with HPV, as it is assumed that the first sexual contact would be with girls of the about the same age without HPV infection (0,5% of population). | [16-18] |
| 7. | HPV type-specific prevalence in different cancers is based on literature. | [19-24] |
| 8. | Vaccine price in base case is set to approximately 50% discount on the list price, is assumed assuming a tender-based pricing. This price is comparable to low-tier European pricing. | [6-8] |
| 9. | Average utilities and survival rates are used for the different types of cancer. No differentiation was implied for different cancer stages. The overall average was calculated. |  |
| 10. | There is no selection in who is getting vaccinated, no specific risk groups are assumed in vaccination strategy. Vaccination is at random. |  |
| 11. | Cancer incidence was calculated by taking the average of cases over the last 5 years, in all male and female cancers. | [25] |
| 12. | One can only be infected by HPV through sexual contact, and thus, a virgin cannot be infected by HPV. |  |
| 13. | Once anyone in the model becomes sexually active (virgin > susceptible), they remain in the “susceptible” health state. |  |
| 14. | We did not explicitly model sexual behaviour and sexual mixing. As we focus on a population base, we did not have separate analysis for Men who have Sex with Men (MSM) or targeted approached for high-risk group vaccination. Also, no transgenders were specifically taken into account. |  |

CIN3+, cervical intraepithelial neoplasia of grade 3 or worse; HPV, human papillomavirus; MSM, Men who have Sex with Men

## **Supplementary Table S4 Model input variables**

| **Transition probability** | **Value** | **Substantiation** (including source) |
| --- | --- | --- |
| Virgin 🡪 Susceptible  Virgin vaccinated 🡪 Vaccinated | Age- / Cycle-dependent  12-15: 0.074  16- 19: 0.18  20-24: 0.19  25-40: 0.13  40+: 0 | Chance to lose virginity and become susceptible for infection. [17, 18]. |
| Virgin 🡪 Vaccinated virgin  Susceptible 🡪 Vaccinated  Infected 🡪 Infected vaccinated | 0.3 | Cycle 0, chance to be vaccinated, corresponds with female vaccination coverage multiplied by proportion of males expected to be vaccinated [13]. |
| Virgin vaccinated 🡪 Virgin  Vaccinated 🡪 Susceptible | 0.0 | Vaccine waning, assumed to be 0, since antibody titers remain high [11, 12]. |
| Susceptible 🡪 Infected | Age- / Cycle- / Type-dependent (Min-Max)  HPV-16: 0.0000003721 – 0.0329  HPV-18: 0 – 0.00917  HPV-31: 0.00002 – 0.012  HPV-33: 0.0000000033 – 0.0046  HPV-45: 0.000000004 – 0.0063  HPV-52: 0.000000001 – 0.0073  HPV-58: 0.000000004 – 0.0072 | The chance to be infected by a specific type of HPV. All transmission probabilities are type-specific. Also, shown in figure 3 [27]. |
| Infected 🡪 Susceptible  Infected vaccinated 🡪 Vaccinated | Type-specific  HPV-16: 0.38  HPV-18: 0.72  Other HrHPV: 0.69 | Chance to clear current infection [28]. |
| Herd effect (% male cancer left after female vaccination) | 0.72 | Effect of 45.5% female vaccination to the male cancer incidence, 72% of cancers are not prevented by female vaccination in males [15]. |
| Infected 🡪 Pre-stage I | 0.048 | Chance to develop a pre-stage of one of the cancers. CIN-specific progression rates are used from previous published models [29, 30]. |
| Pre-stage I 🡪 Pre-stage II | 0.1113 | Chance to progress from pre-stage I to pre-stage II. CIN progression rates used from previous published models [29, 30]. |
| Pre-stage I 🡪 Susceptible | 0.375 | Chance to clear Pre-stage I cancer and become susceptible [29, 30]. |
| Pre-stage II 🡪 Pre-stage III | 0.0469 | Chance to progress from pre-stage II to pre-stage III. CIN progression rates used from previous published model [29, 30]. |
| Pre-stage II 🡪 Susceptible | 0.02 | Chance to clear Pre-stage II cancer and become susceptible [29, 30]. |
| Pre-stage III 🡪 Cancer | Fit to real-world incidence data, Calibrated in model. | The chance to develop ano- genital- oropharyngeal- cancer. Model fitted to observed incidence data in the Netherlands. |
| Pre-stage III 🡪 Susceptible | 0.02 | Chance to clear Pre-stage III cancer and become susceptible [29, 30]. |
| Anal cancer 🡪 Anal cancer mortality | From stage:  Anal Cancer I: 0.18  Anal Cancer II: 0.1148  Anal Cancer III: 0.0478  Anal Cancer IV: 0.0183  Anal Cancer V: 0.01163  Survival: 0.01 | Chance to die due to anal cancer, divided into 5 stages with corresponding mortality rates to real-world cancer mortality in the Netherlands [25]. |
| Anal cancer V 🡪 Anal cancer survival | 0.9817 | Transition to a post-anal-cancer state [31]. |
| Penile cancer 🡪 Penile cancer mortality | From stage:  Penile Cancer I: 0.1  Penile Cancer II: 0.063  Penile Cancer III: 0.025  Penile Cancer IV: 0.008  Penile Cancer V: 0.0158  Survival: 0.01 | Chance to die due to penile cancer, divided into 5 stages with corresponding mortality rates to real-world cancer mortality in the Netherlands [25]. |
| Penile cancer V🡪 Penile cancer survival | 0.9842 | Transition to a post-penile-cancer state [31]. |
| Oropharyngeal cancer V 🡪 Oropharyngeal cancer mortality | From stage  Oropharyngeal Cancer I: 0.31  Oropharyngeal Cancer II: 0.0966  Oropharyngeal Cancer III: 0.0392  Oropharyngeal Cancer IV: 0.0196  Oropharyngeal Cancer V: 0.0135  Survival 0.03 | Chance to die due to oropharyngeal cancer, divided into 5 stages with corresponding mortality rates to real-world cancer mortality in the Netherlands [25]. |
| Oropharyngeal cancer V 🡪 Oropharyngeal cancer survival | 0.9865 | Transition to a post-oropharyngeal-cancer state [31]. |
| Any health-stage 🡪 General mortality | Age- / cycle-dependent (min-max) 12-99 years old.  (0.00011 – 0.3815) | Dutch general mortality rate derived from Dutch specific numbers [31]. |

CIN, cervical intraepithelial neoplasia, HPV, human papillomavirus

**Figure S1 Deterministic sensitivity analysis**

*
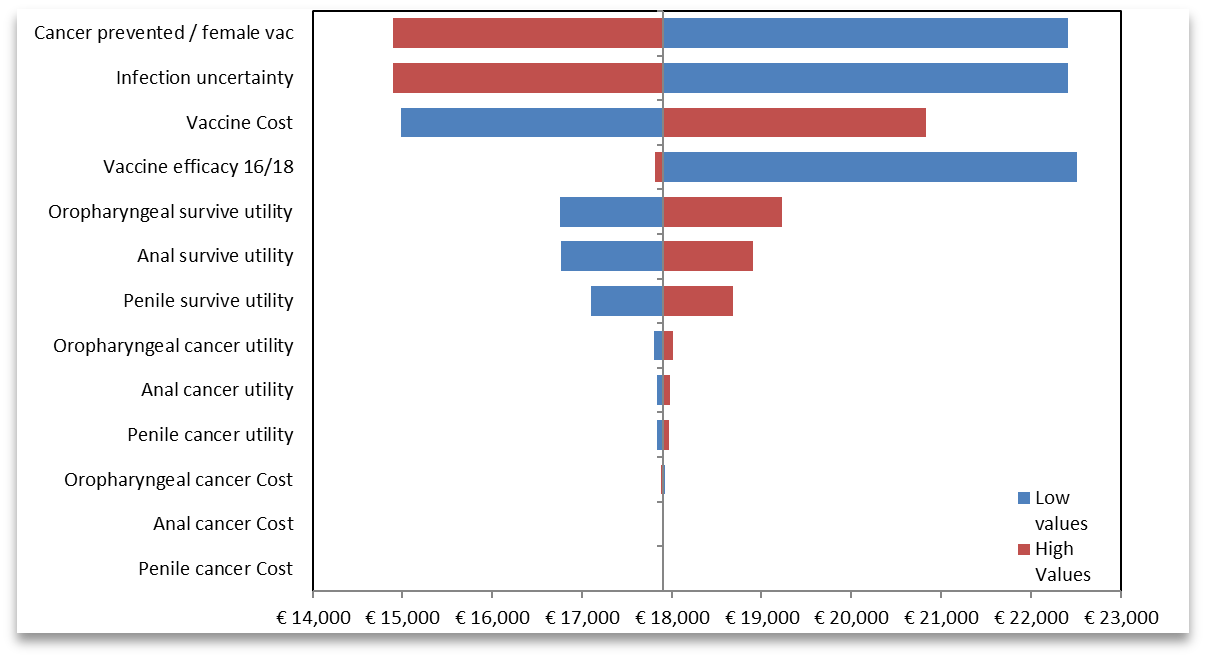
*


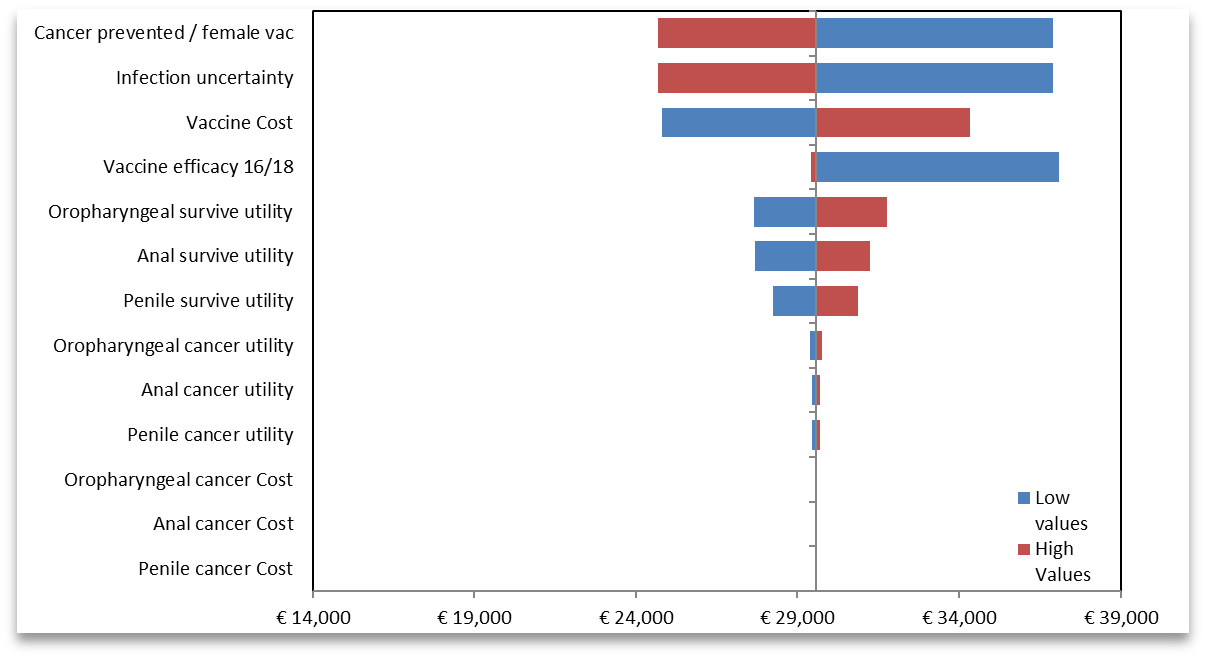


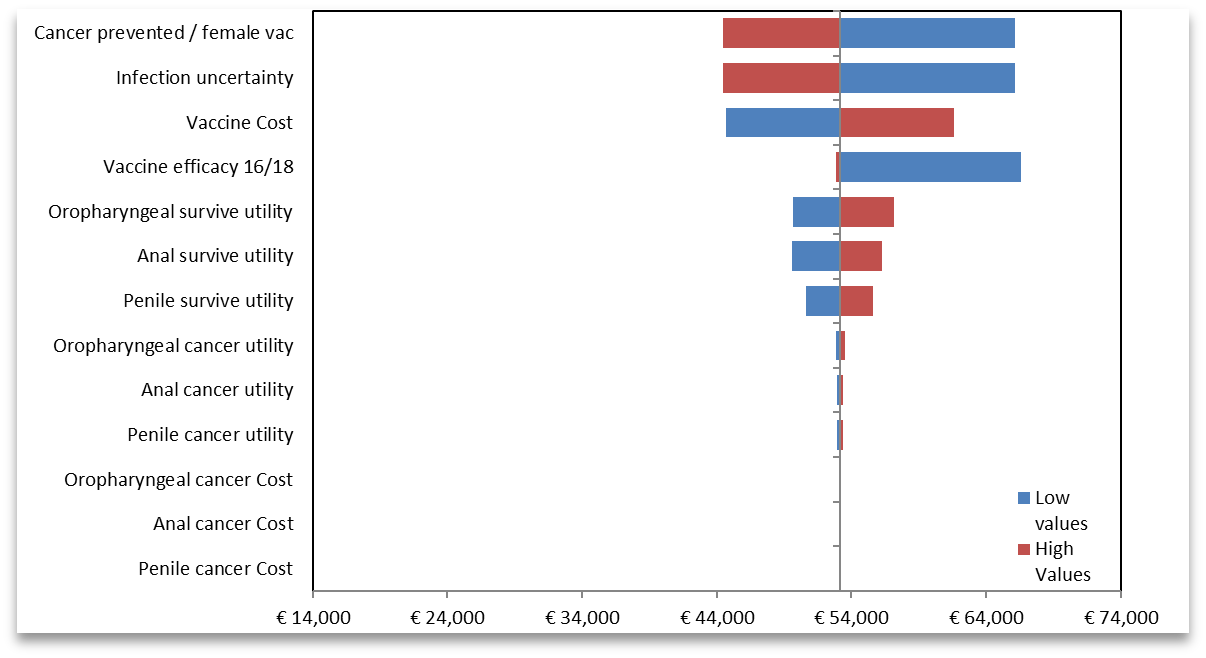


*Three separate deterministic sensitivity analyses. Top) 12-year-old; Middle) 18-year-old; Bottom) 26-year-old.*

**Figure S2 Probabilistic sensitivity analysis**

*
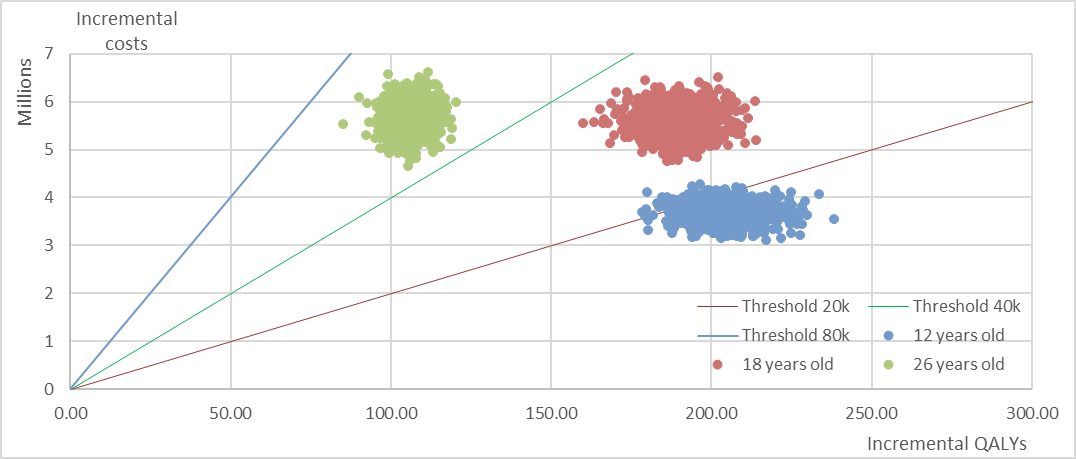
*

*Three separate probabilistic sensitivity analyses.*

## **Supplementary table S5. Input parameters varied in the Deterministic sensitivity analysis**

| Parameter | Base case value | Lower bound value | Upper bound value |
| --- | --- | --- | --- |
| Cancer prevented by female vaccination | 72% | 58% | 86% |
| Vaccine costs per dose | €50 | €40 | €60 |
| Vaccine efficacy vs types 16 and 18 | 99.5% | 79.6% | 100% |
| Anal cancer cost | €5460.25 | €4368.20 | €6552.30 |
| Penile cancer cost | €4368.20 | €3494.56 | €5241.84 |
| Oropharyngeal cancer cost | €6552.30 | €5241.84 | €7862.76 |
| Anal cancer utility | 0.57 | 0.45 | 0.67 |
| Penile cancer utility | 0.79 | 0.65 | 0.97 |
| Oropharyngeal cancer utility | 0.58 | 0.49 | 0.74 |
| Anal survive utility | 0.87 | 0.66 | 0.99 |
| Penile survive utility | 0.85 | 0.65 | 0.98 |
| Oropharyngeal survive utility | 0.75 | 0.58 | 0.87 |
| Vaccine coverage | 30% | 24% | 36% |
| Infection uncertainty | 100% | 80% | 120% |

## **Figure S3. Plain Language Summary**


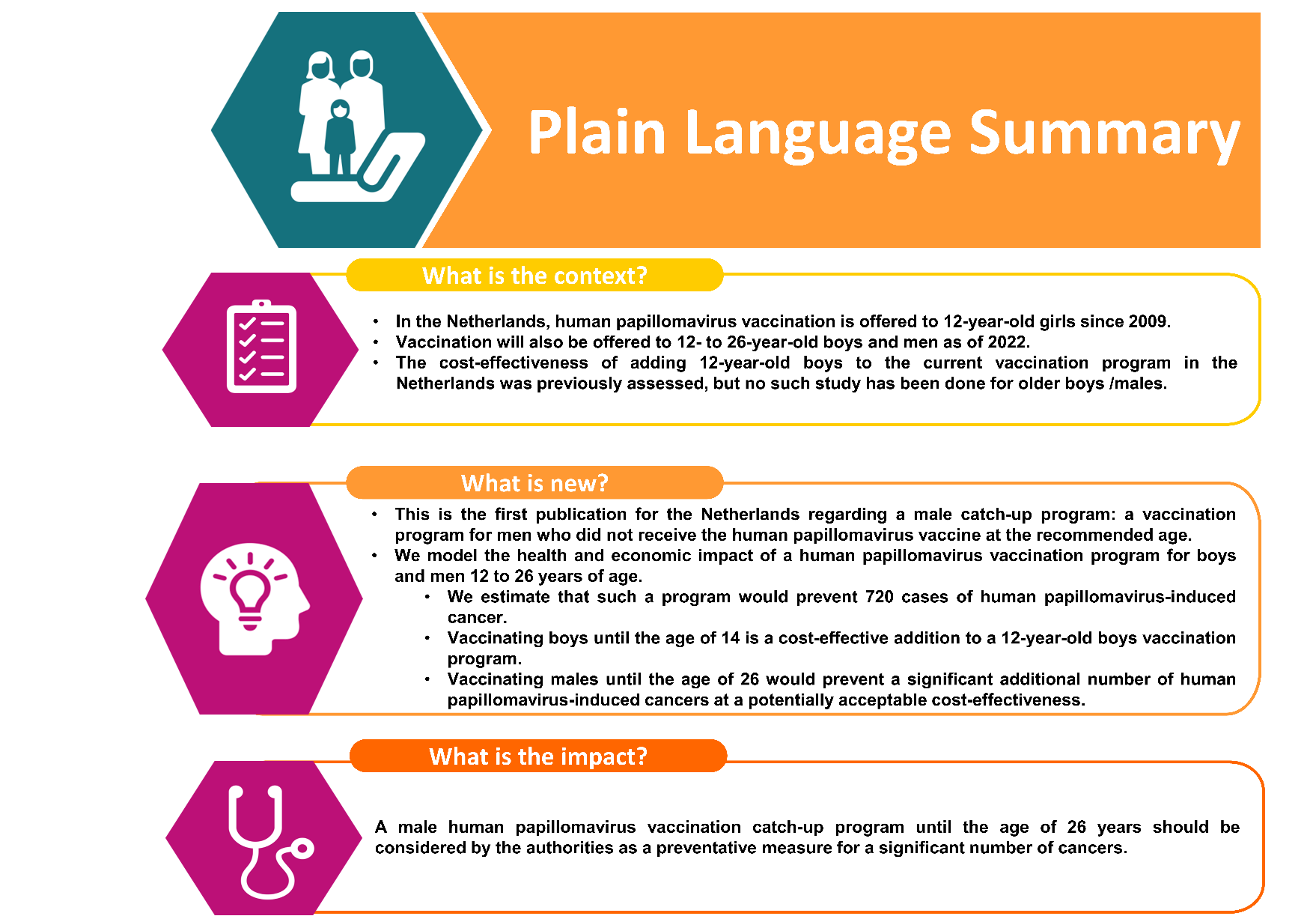


## **Supplementary materials references**

[1] Heijink R, van Baal P, Oppe M, Koolman X, Westert G. Decomposing cross-country differences in quality adjusted life expectancy: the impact of value sets. Popul Health Metr. 2011;9(1):17.

<https://doi.org/10.1186/1478-7954-9-17>

[2] Burger EA, Sy S, Nygard M, Kristiansen IS, Kim JJ. Prevention of HPV-related cancers in Norway: cost-effectiveness of expanding the HPV vaccination program to include pre-adolescent boys. PLoS One. 2014;9(3):e89974.

<https://doi.org/10.1371/journal.pone.0089974>

[3] Pearson AL, Kvizhinadze G, Wilson N, Smith M, Canfell K, Blakely T. Is expanding HPV vaccination programs to include school-aged boys likely to be value-for-money: a cost-utility analysis in a country with an existing school-girl program. BMC Infect Dis. 2014;14(1):351.

<https://doi.org/10.1186/1471-2334-14-351>

[4] Blakely T, Kvizhinadze G, Karvonen T, Pearson AL, Smith M, Wilson N. Cost-effectiveness and equity impacts of three HPV vaccination programmes for school-aged girls in New Zealand. Vaccine. 2014;32(22):2645-56.

<https://doi.org/10.1016/j.vaccine.2014.02.071>

[5] Chesson HW, Ekwueme DU, Saraiya M, Dunne EF, Markowitz LE. The cost-effectiveness of male HPV vaccination in the United States. Vaccine. 2011;29(46):8443-50.

<https://doi.org/10.1016/j.vaccine.2011.07.096>

[6] Slovakia Government. Health care and intervention costs for the Slovakia Governement. 2017.

<https://www.liekinfo.sk/lieky.aspx> (accessed 23 August 2019).

[7] Rijksinstituut voor Ziekte- en Invaliditeitsverzekering. Institut national d'assurance maladie-invalidité. Kosten medische interventisch en medicatie België. 2018.

<http://ondpanon.riziv.fgov.be/SSPWebApplicationPublic/nl/Public/ProductSearch> (accessed 23 August 2019).

[8] Luxembourg. Luxembourg medication costs. 2015.

<https://cns.public.lu/en/legislations/textes-coordonnes/liste-med-comm.html> (accessed 23 August 2019).

[9] Hakkart-van Roijen L, van der Linden N, Bouwmans C, Kanters T, Tan SS. Kostenhandleiding: Methodologie van kostenonderzoek en referentieprijzen voor economische evaluaties in de gezondheidszorg. Institute for Medical Technology Assessment, Erasmus Universiteit Rotterdam. 2016.

<https://www.zorginstituutnederland.nl/publicaties/publicatie/2016/02/29/richtlijn-voor-het-uitvoeren-van-economische-evaluaties-in-de-gezondheidszorg> (accessed 23 August 2019).

[10] de Kok IM, Habbema JD, van Rosmalen J, van Ballegooijen M. Would the effect of HPV vaccination on non-cervical HPV-positive cancers make the difference for its cost-effectiveness? Eur J Cancer. 2011;47(3):428-35.

<https://doi.org/10.1016/j.ejca.2010.09.030>

[11] Qendri V, Bogaards JA, Berkhof J. Health and Economic Impact of a Tender-Based, Sex-Neutral Human Papillomavirus 16/18 Vaccination Program in the Netherlands. J Infect Dis. 2017;216(2):210-219.

<https://doi.org/10.1093/infdis/jix272>

[12] Suijkerbuijk AW, Donken R, Lugner AK, de Wit GA, Meijer CJ, de Melker HE, et al. The whole story: a systematic review of economic evaluations of HPV vaccination including non-cervical HPV-associated diseases. Expert Rev Vaccines. 2017;16(4):361-375.

<https://doi.org/10.1080/14760584.2017.1256778>

[13] Rijksinstituut voor Volksgezondheid en Milieu. Vaccinatiegraad en jaarverslag Rijksvaccinatieprogramma Nederland 2017/18. Available from: [The National Immunisation Programme in the Netherlands (rivm.nl)](https://www.rivm.nl/bibliotheek/rapporten/2018-0124.pdf) <https://doi.org/10.21945/rivm-2017-0010>

[14] Lehtinen M, Paavonen J, Wheeler CM, Jaisamrarn U, Garland SM, Castellsagué X, et al. Overall efficacy of HPV-16/18 AS04-adjuvanted vaccine against grade 3 or greater cervical intraepithelial neoplasia: 4-year end-of-study analysis of the randomised, double-blind PATRICIA trial. Lancet Oncol. 2012;13(1):89-99.

<https://doi.org/10.1016/S1470-2045(11)70286-8>

[15] Bogaards JA, Wallinga J, Brakenhoff RH, Meijer CJ, Berkhof J. Direct benefit of vaccinating boys along with girls against oncogenic human papillomavirus: bayesian evidence synthesis. BMJ. 2015;350:h2016.

<https://doi.org/10.1136/bmj.h2016>

[16] de Looze M, van Dorsselaer S, de Roos S, Verdurmen J, Stevens G, Gommans R, et al. HBSC 2013. Gezondheid, welzijn en opvoeding van jongeren in Nederland Nederland [Health, well-being, and upbringing of adolescents in the Netherlands]. In: Planbureau SeC, editor. Utrecht2013. <https://dspace.library.uu.nl/bitstream/handle/1874/304333/HBSC_NL_Rapport_2013.pdf?sequence=1&isAllowed=y> (accessed 23 August 2019). Universiteit Utrecht;2014.

[17] de Graaf H, Meijer S, Poelman J. Seks onder je 25e : Seksuele gezondheid van jongeren in Nederland anno 2005. Delft : Eburon <https://eburon.nl/product/seks_onder_je_25e/> (accessed 23 August 2019).

[18] Bakker F, Vanwesenbeeck I. Seksuele gezondheid in Nederland. Huisarts en Wetenschap. 2007;50(5):372.

<https://doi.org/10.1007/BF03085175>

[19] Backes DM, Kurman RJ, Pimenta JM, Smith JS. Systematic review of human papillomavirus prevalence in invasive penile cancer. Cancer Causes Control. 2009;20(4):449-57.

<https://doi.org/10.1007/s10552-008-9276-9>

[20] De Vuyst H, Clifford GM, Nascimento MC, Madeleine MM, Franceschi S. Prevalence and type distribution of human papillomavirus in carcinoma and intraepithelial neoplasia of the vulva, vagina and anus: a meta-analysis. Int J Cancer. 2009;124(7):1626-36.

<https://doi.org/10.1002/ijc.24116>

[21] Hoots BE, Palefsky JM, Pimenta JM, Smith JS. Human papillomavirus type distribution in anal cancer and anal intraepithelial lesions. Int J Cancer. 2009;124(10):2375-83.

<https://doi.org/10.1002/ijc.24215>

[22] Kreimer AR, Chaturvedi AK. HPV-associated Oropharyngeal Cancers--Are They Preventable? Cancer Prev Res (Phila). 2011;4(9):1346-9.

<https://doi.org/10.1158/1940-6207.CAPR-11-0379>

[23] Miralles-Guri C, Bruni L, Cubilla AL, Castellsague X, Bosch FX, de Sanjose S. Human papillomavirus prevalence and type distribution in penile carcinoma. J Clin Pathol. 2009;62(10):870-8.

<https://doi.org/10.1136/jcp.2008.063149>

[24] Rietbergen MM, Leemans CR, Bloemena E, Heideman DA, Braakhuis BJ, Hesselink AT, et al. Increasing prevalence rates of HPV attributable oropharyngeal squamous cell carcinomas in the Netherlands as assessed by a validated test algorithm. Int J Cancer. 2013;132(7):1565-71.

<https://doi.org/10.1002/ijc.27821>

[25] Integraal Kankercentrum Nederland. Cijfers over kanker. 2017.

<https://www.cijfersoverkanker.nl> (accessed 23 August 2019).

[26] Barnabas R. Deterministic compartmental models application: Application: Modeling the Potential Benefit of HPV Vaccines. 2012.

<http://www.scharp.org/pdf_files/VIDI/meeting/Barnabas_HPV_Model.pdf> (accessed 23 August 2019).

[27] Luttjeboer J, Westra TA, Wilschut JC, Nijman HW, Daemen T, Postma MJ. Cost-effectiveness of the prophylactic HPV vaccine: an application to the Netherlands taking non-cervical cancers and cross-protection into account. Vaccine. 2013;31(37):3922-7.

<https://doi.org/10.1016/j.vaccine.2013.06.044>

[28] Giuliano AR, Palefsky JM, Goldstone S, Moreira ED, Jr., Penny ME, Aranda C, et al. Efficacy of quadrivalent HPV vaccine against HPV Infection and disease in males. N Engl J Med. 2011;364(5):401-11.

<https://doi.org/10.1056/NEJMoa0909537>

[29] Rogoza RM, Ferko N, Bentley J, Meijer CJ, Berkhof J, Wang KL, et al. Optimization of primary and secondary cervical cancer prevention strategies in an era of cervical cancer vaccination: a multi-regional health economic analysis. Vaccine. 2008;26 Suppl 5:F46-58.

<https://doi.org/10.1016/j.vaccine.2008.02.039>

[30] Rogoza RM, Westra TA, Ferko N, Tamminga JJ, Drummond MF, Daemen T, et al. Cost-effectiveness of prophylactic vaccination against human papillomavirus 16/18 for the prevention of cervical cancer: adaptation of an existing cohort model to the situation in the Netherlands. Vaccine. 2009;27(35):4776-83.

<https://doi.org/10.1016/j.vaccine.2009.05.085>

[31] Centraal Bureau Statistiek. General Mortality Rates for every age. 2017.

<http://statline.cbs.nl/StatWeb/publication/?PA=70895ned> (accessed 23 August 2019).
